# Gallbladder Tuberculosis Mimicking Gallbladder Carcinoma: A Case Report and Review of the Literature

**DOI:** 10.7759/cureus.7950

**Published:** 2020-05-04

**Authors:** Abdelbassir Ramdani, Obed Rockson, Tariq Bouhout, Badr Serji, Tijani El Harroudi

**Affiliations:** 1 Surgical Oncology, Mohammed VI University Hospital, Regional Oncology Center, Oujda, MAR

**Keywords:** gallbladder tuberculosis, tuberculosis, gallbladder carcinoma, cholecystectomy, surgery, case report, scare

## Abstract

Gallbladder tuberculosis (GT) is an extremely rare disease entity, even in our country Morocco known for being an endemic area. The lack of pathognomonic clinical presentation and radiological features of GT makes preoperative diagnosis unlikely and poses a diagnostic dilemma regarding gallbladder carcinoma (GC). The diagnosis is usually made by histological examination after cholecystectomy, highlighting the importance of sending every gallbladder specimen to pathology. We report an exceedingly rare case of GT mimicking GC and refer to a recent review of the literature to discuss the clinical and radiological features of GT.

## Introduction

Gallbladder tuberculosis (GT) is an extremely rare clinical entity that represents only 1% of abdominal tuberculosis cases [1]. The clinical presentation and the radiological findings are not specific, thereby making preoperative diagnosis difficult, and it is usually misdiagnosed as gallbladder carcinoma (GC) [2]. The diagnosis is usually made upon histological examination after cholecystectomy. We report an uncommon case of GT mimicking GC, and refer to a recent review of the literature to discuss the clinical and radiological features of GT. This case has been reported following the SCARE criteria [3].

## Case presentation

**Figure 1:**
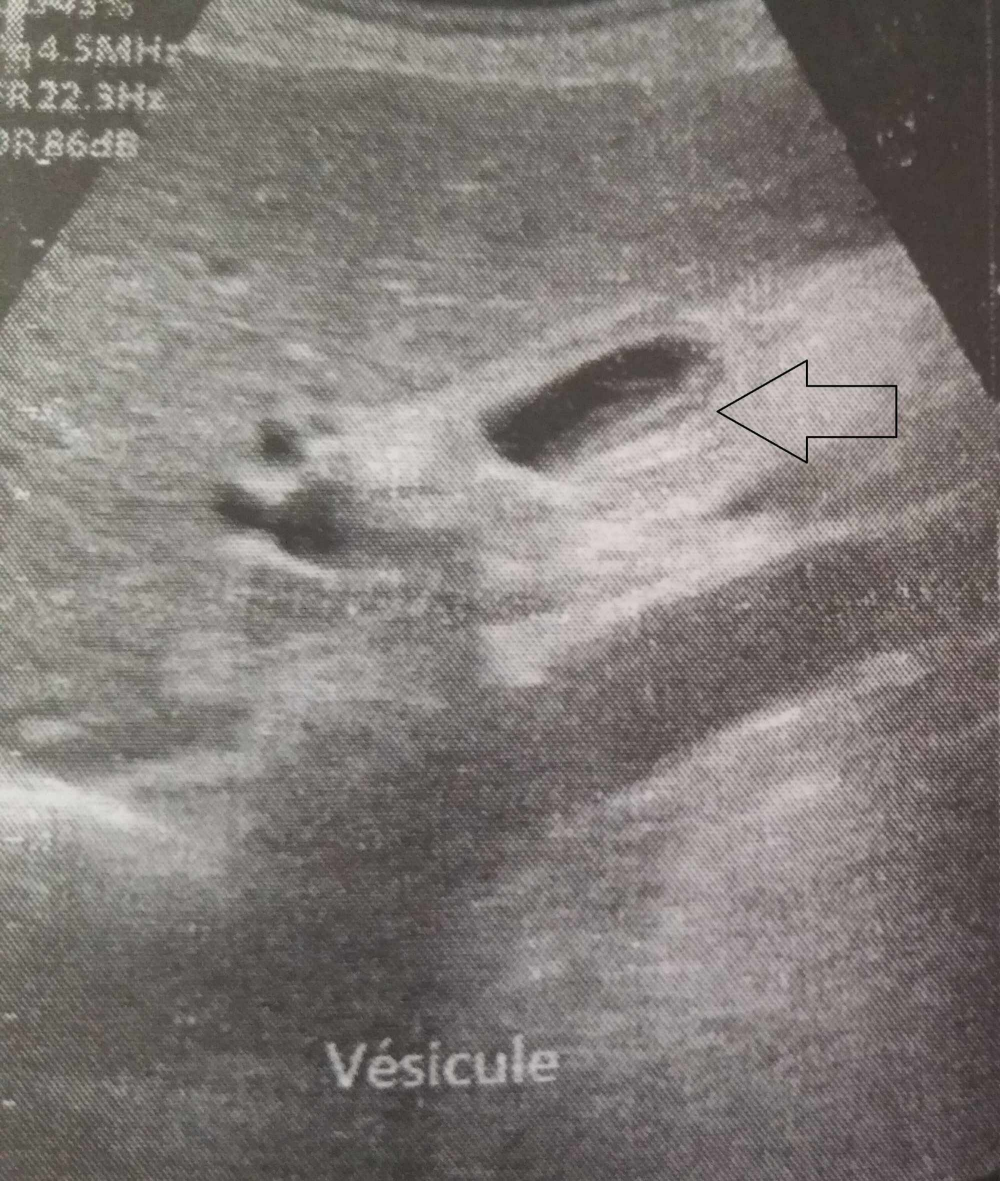
Ultrasonography showing thickened gallbladder wall (black arrow).

A 71-year-old male patient, a former miner, was diagnosed with silicosis 30 years ago. The patient presented with complaints of vague, dull pain in the right upper quadrant with a significant loss of weight and appetite. There was no history of tuberculosis. Physical examination found a right hypochondrial tenderness. Laboratory investigations and chest X-rays were normal. Ultrasonography showed a gallbladder with a large gallstone and an irregularly thickened wall with dilatation of the intrahepatic bile ducts (IHBD) and the common bile duct (CBD) (Figure 1).

**Figure 2:**
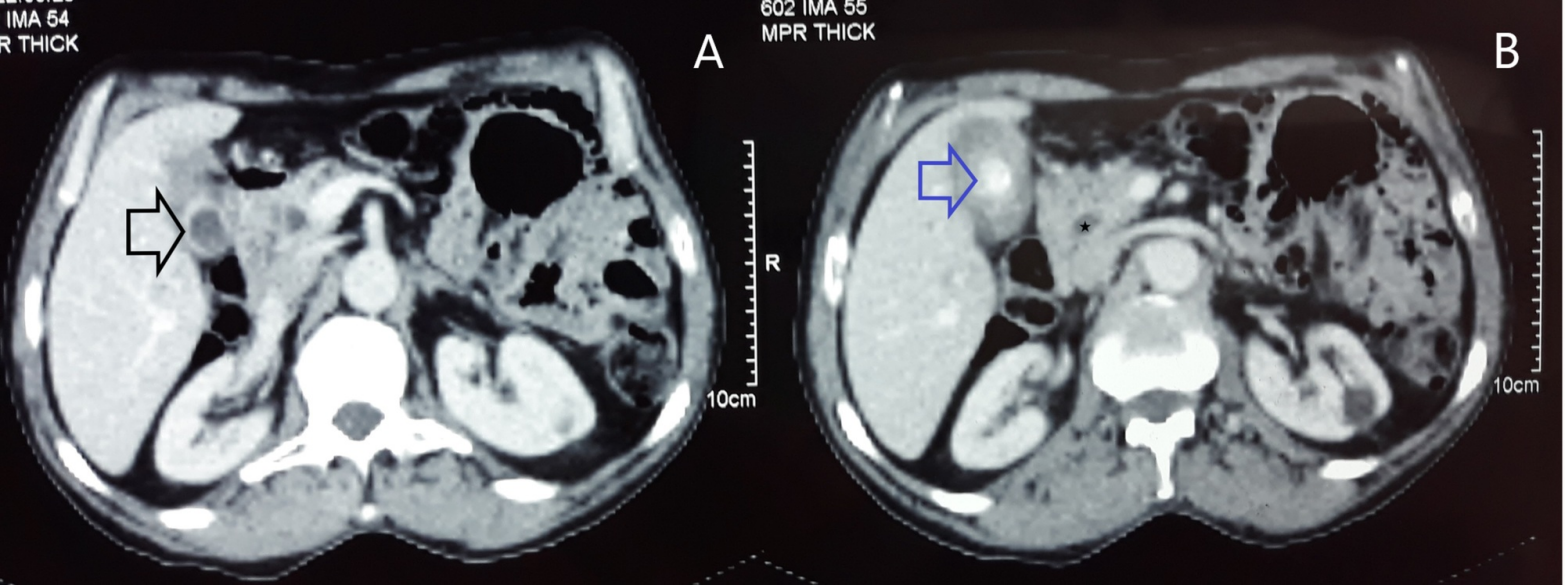
(A and B) Computed tomography scan showing irregular thickening of the gallbladder wall (black arrow) with the presence of a large calculus in the gallbladder (blue arrow), with dilatation of the common bile duct (star).

A CT scan with intravenous contrast reported a suspicious irregular thickening of the gallbladder wall with the presence of a large calculus of 18 mm in the gallbladder, with dilatation of the CBD (Figure 2A,B).

**Figure 3:**
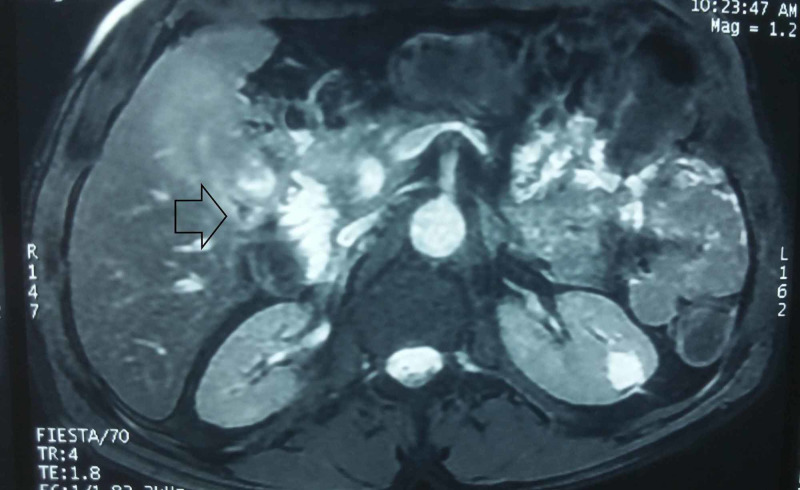
Bili-Magnetic resonance imaging showing suspicious irregular thickening of the gallbladder wall (black arrow).

Bili-MRI also revealed a thickened irregular gallbladder wall with dilatation of the CBD (14 mm) and IHBD, making GC the most likely diagnosis (Figure 3).

**Figure 4:**
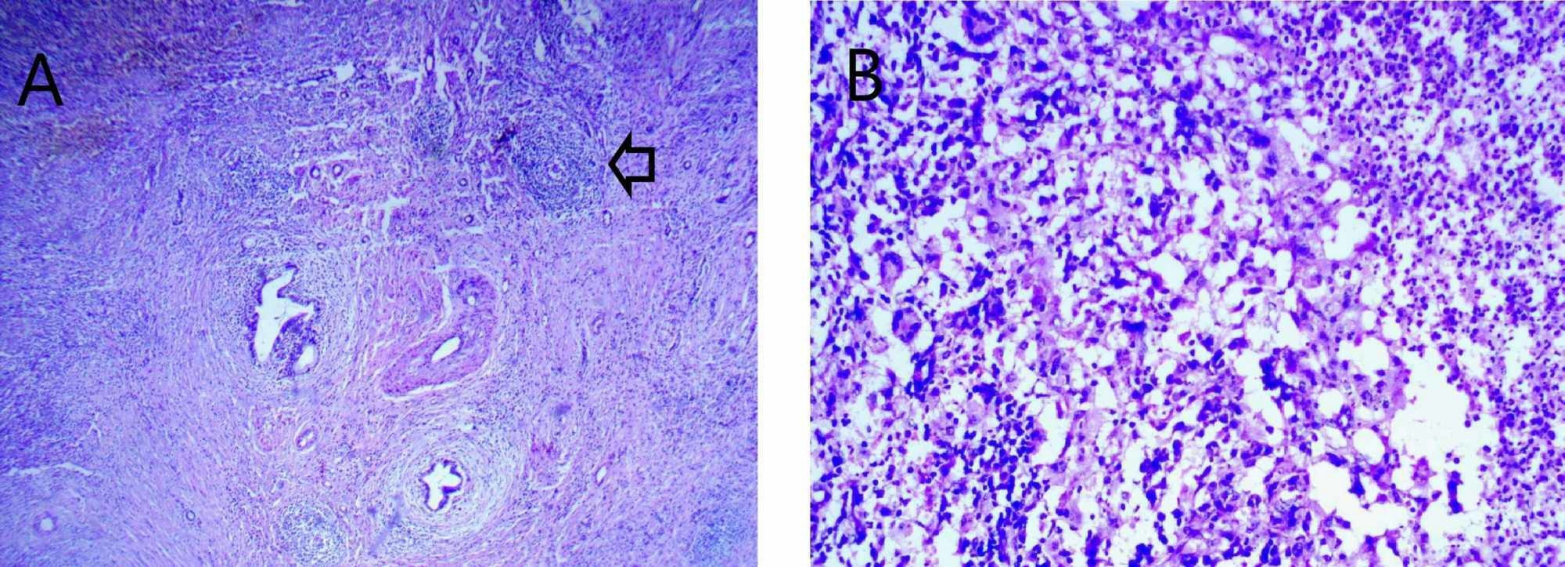
(A) Microphotograph showing an epithelioid granuloma (black arrow) (HE 40×). (B) Microphotograph showing an epithelioid cell granuloma with rare giant cells without caseous necrosis (HE 400×). HE: Hematoxylin and eosin

Surgical exploration of the presumptive diagnosis of GC was done. Intraoperatively, we found a hard gallbladder with a thickened wall and contiguous infiltration into segment 4b of the liver. The patient underwent radical cholecystectomy. The histopathological examination revealed an ulcerated mucosa surrounded by epithelioid cells mixed with rare giant Langhans cells without caseous necrosis (Figure 4A,B). 

The diagnosis of GT was established in front of a bundle of clinical, radiological, and histological arguments.

On postoperative day nine, the patient became febrile and complained of abdominal pain and vomiting. An abdominal examination revealed diffuse rebound tenderness. Bile was noted in the drain. An urgent abdominal CT objectified fluid collections in the perihepatic space and at the cul-de-sac of Douglas, suggesting biliary peritonitis. The patient benefited from lavage and drainage of the peritoneal cavity and had a satisfactory recovery before being discharged from the hospital on day 15.

A complete course of anti-tubercular treatment was administered for six months [four drugs for two months: isoniazid (5 mg/kg), rifampicin (10 mg/kg), pyrazinamide (25 mg/kg), and ethambutol (20 mg/kg), and then two drugs for the next four months: isoniazid and rifampicin]. The patient remained asymptomatic after a six-month follow-up.

## Discussion

Isolated GT is an extremely rare clinical entity that was originally described for the first time by Gaucher in 1870 [4]. Only around 120 cases have been reported in the literature [1]. GT occurs most commonly in women over 30 years of age [1, 5]. Cholelithiasis and cystic duct obstruction are considered the most important factors in the development of GT [6]. According to Kapoor et al., all patients presented gallstone disease [5], however, Saluja et al. did not find any gallstone disease associated with GT in their cases [7].

The rarity of tuberculosis in the gallbladder is possibly due to the hypovascularity of the gallbladder sac and the alkaline nature of gallbladder bile, which has an inhibitory effect on Mycobacterium [8].

The clinical presentation of GT is varied and nonspecific, making the preoperative diagnosis of GT difficult; the most frequent clinical signs and symptoms are abdominal pain, loss of weight, and loss of appetite, according to the series of GT published in the literature, as seen in our case (Table 1).

**Table 1:** Comparison of the symptoms and clinical signs of GT between the series published in the literature and our case. GT, gallbladder tuberculosis

Author (year)	Kumar et al. (2000) [9]	Kapoor et al. (2006) [5]	Saluja et al. (2007) [7]	Govindasamy et al. (2011) [10]	Xu et al. (2011) [1]	Krishnamurthy et al. (2016) [2]	Our case
Number of patients	5	5	3	3	7	3	1
Abdominal pain	5	4	2	3	7	3	1
Loss of weight and appetite	1	1	1	2	3	2	1
Fever	1	0	1	2	2	1	0
Abdominal mass	1	1	1	1	1	0	0
Jaundice	0	0	0	2	0	0	0

Only a few papers on the radiological findings of GT are found in the literature. Xu et al. [1] revealed three types of CT morphology features of GT. The most common presentation is the thickened-wall type which can be frequently misdiagnosed as GC, or cholecystitis. Sometimes CT scans can differentiate between GT and GC by showing the “halo” of the edema of GT. The mass-forming type CT findings are similar to GC; however, the presence of a large mass with multicentric necrosis on enhanced CT, or a large mass with multiple flecked calcifications, may be helpful to make the diagnosis of GT. The micronodular type is nonspecific.

According to Govindasamy et al., the MRI morphology features of GT were identical to GC [10]. To summarize, there are no specific investigations for GT.

The diagnosis is usually made upon histological examination after cholecystectomy [11] highlighting the importance of sending every gallbladder specimen to pathology.

The treatment of GT is based on anti-tubercular chemotherapy based on a two-month attack treatment plan involving isoniazid (5 mg/kg), rifampicin (10 mg/kg), pyrazinamide (25-30 mg/kg), and ethambutol (20 mg/kg), followed by maintenance therapy for four months of isoniazid and rifampicin [12].

## Conclusions

There is no pathognomonic presentation of GT, and it is usually misdiagnosed as GC. As the preoperative diagnosis is difficult, all resected cholecystectomy specimens should be sent for histopathological examination for evidence of tuberculosis. A well-managed anti-tuberculosis treatment allows healing.
